# Enhanced deep learning model for predicting hydraulic performance in recycled porous pipe irrigation systems

**DOI:** 10.1038/s41598-025-20354-6

**Published:** 2025-10-01

**Authors:** Mohamed Ahmed Moustafa, Ahmed Amin, Zaharaddeen Aminu Bello, Khaled A. M. Ali, Hassan A. A. Sayed, Yasser Kamal Osman, Sherouk Hassan, Muhammad Aurangzaib, Mostafa. H. Fayed

**Affiliations:** 1 Faculty of Agricultural Engineering, Al-Azhar University, Cairo, 11751 Egypt; 2 College of Engineering, Nanjing Agricultural University, Nanjing, 210031 China; 3 College of Economics and Managment, Nanjing Agricultural University, Nanjing, 210095 China; 4 Agricultural Engineering Research Institute (AEnRI), Agricultural Research Center (ARC), Giza, 12311 Egypt; 5 School of Energy and Environment Science, Yunnan Provincial Rural Energy Engineering Key Laboratory, Yunnan Normal University, Chenggong University Town, No. 768 Juxian Road, Kunming, P.R., 650500 China

**Keywords:** Hydraulic evaluation, Recycled porous pipes, Sustainable irrigation, Deep learning, Water conservation, Environmental sciences, Engineering

## Abstract

This study evaluates the hydraulic performance of irrigation systems using recycled porous pipes and the predictive modeling capabilities of deep learning algorithms for discharge rates, comparing Type A (recycled rubber-polyethylene blend) and Type B (recycled rubber only). Laboratory experiments measured discharge rates, coefficient of variation (CV), and emission uniformity (EU) across pressures (20–80 kPa) and pipe lengths (3–9 m). Results showed strong discharge-pressure correlations (R^2^ = 0.95–0.97). Type B achieved superior performance at 80 kPa, with lower CV (8.80%) and higher EU (87.25%) versus Type A (CV = 9.54%, EU = 84.60%), indicating enhanced flow efficiency. Statistical analysis confirmed significant differences (p < 0.05) between pipe types. Four deep learning models—Enhanced Multilayer Perceptron (MLP), Long Short-Term Memory (LSTM), Deep Neural Network (DNN), and Artificial Neural Network (ANN)—were developed to predict discharge rates based on pressure, pipe length, and material type. Synthetic data augmentation (GANs) was used to overcome limited experimental samples.The Enhanced MLP mode achieved the highest predictive accuracy (R^2^ = 0.9891, RMSE = 0.2762), outperforming all other models.This integration of hydraulic evaluation and AI modeling supports real-time irrigation scheduling, enhances water efficiency in water-scarce regions, and highlights the critical influence of material choice.

## Introduction

The growing global demand for freshwater resources, fueled by population increase, urbanization, and the mounting effects of climate change, poses a severe challenge to sustainable agricultural practices. Agriculture, the world’s greatest consumer of freshwater, currently accounts for around 70% of total withdrawals, with a projected 75% by 2025^1–4^. This increasing demand for water resources, combined with the fact that over two billion people globally already face water scarcity^5–8^ highlights the critical need for new and effective irrigation systems. Subsurface irrigation (SSI), particularly when using porous pipes, has emerged as a potential strategy for delivering water directly to the root zone, reducing water losses from evaporation and surface runoff by up to 70% compared to traditional methods. This method is especially important in arid and semi-arid countries with limited water and energy resources^9–11^. Studies have shown that SSI systems can reach water utilization efficiencies of up to 95%, well above the 75–85% efficiency generally seen in traditional surface irrigation systems^12–14^.

Porous pipes, which include both perforated and drop lines, are vital components of SSI systems. By allowing water to be released into the soil profile gradually and carefully, these pipes help to minimize wasteful water loss and maintain constant moisture levels in the plant root zone. Porous pipe systems can lower water application rates by up to 50% when compared to conventional irrigation methods like sprinklers, which makes them an attractive option for encouraging sustainable farming methods and boosting water conservation initiatives^15–18^. Furthermore, the incorporation of recycled materials, along with rubber and polyethylene, in the manufacturing of these pipes aligns with broader sustainability aims by lowering waste era and selling rounds^19–21^.

Porous pipes are usually categorized according to the materials that make them up. Two varieties are the material of this study: Type A, which is made entirely of recycled rubber, and Type B, which is made of a combination of recycled rubber and polyethylene. The addition of polyethylene to Type A pipes typically results in increased tensile strength and flexibility, which enhances their longevity and adaptability under a variety of irrigation settings^22^. In terms of manufacturing procedures, this material combination also provides benefits, enabling constant product quality and dependable hydraulic performance. On the other hand, Type B pipes put environmental concerns first by using waste materials like used tires, which helps to reduce waste streams and may even lower production costs^23^. Nonetheless, Type B pipes exhibit lower mechanical strength than Type A under high pressures (> 60 kPa) due to the absence of polyethylene reinforcement, which compromises structural integrity^24,25^.

Intelligence algorithms, particularly deep learning (DL), have emerged as powerful tools for extracting meaningful features from complex agricultural data and developing sophisticated decision support systems^26^. These algorithms have demonstrated remarkable success across various agricultural applications. For example, Younes et al.^27^ employed a convolutional neural network (CNN) to model soil moisture from pictures, outperforming traditional machine learning methods such as random forests (RF), support vector machines (SVM), and artificial neural networks (ANN)^28^. Employed a multilayer perceptron (MLP) with deep learning to predict daily evapotranspiration, which beat gradient-boosting machines, random forests, and generalized linear models. Other studies have further validated the effectiveness of deep learning approaches in agricultural contexts. e Lucas et al.^29^ compared three distinct CNN architectures for evapotranspiration prediction and found them to significantly outperform conventional time series models in terms of both accuracy and computational efficiency. Adeyemi et al.^30^ successfully implemented a neural network for soil moisture prediction, achieving remarkable R^2^ values above 0.94 across all test locations. The long short-term memory (LSTM) architecture, a specialized recurrent neural network, has also shown particular promise in handling time-series agricultural data. Alibabaei et al.^31^ demonstrated that a single-layer bidirectional LSTM could achieve R^2^ scores between 0.96 and 0.98 for one-day-ahead predictions of evapotranspiration and soil water content. Furthermore, Alibabaei et al.^32^ found that a Deep Q-Network (DQN) with LSTM models can significantly improve irrigation scheduling. The DQN increased tomato field productivity by 11% while reducing water consumption by 20–30% compared to traditional methods.

Efficient water management in irrigation systems requires predictive models capable of capturing both spatial and temporal variations in system performance. CNN effectively extract spatial patterns from input variables such as pressure, pipe length, and material type, while LSTM networks capture temporal dependencies arising from seasonal changes and fluctuating system loads. Integrating these deep learning approaches enables more accurate discharge predictions, supporting data-driven and adaptive irrigation scheduling^29–31^.

Despite these advances, their application to optimize recycled-material porous pipes remains limited. Predictive modeling in irrigation has predominantly focused on virgin materials or non-recycled composites, overlooking the unique hydraulic characteristics of recycled rubber systems. Furthermore, comparative analyses of distinct recycled compositions (e.g., rubber vs. rubber-polyethylene blends) under standardized conditions are scarce, restricting the development of AI-driven, sustainable irrigation designs.

Several previous studies have focused on the hydraulic performance of porous pipes at different operational parameters, such as pressure and the length of the pipe. As an illustration,^11,14,33–35^ found that the operating pressure has a significant impact on hydraulic performance, as by increasing it, the discharge of these pipes increases and the coefficient of variation (CV) decreases. One of the pioneering studies regarding pressure-discharge relationships in drip irrigation systems, while evaluating the effect of the length of porous pipes on hydraulic performance, was done by^25,36,37^, who found that the length of the pipes affects the hydraulic performance; as the length increases, the discharge decreases. Other studies have evaluated length effects on hydraulic performance^33,34,38^. While previous research has explored porous pipe irrigation and the application of deep learning in agricultural water management, Prior works suffer from key limitations, including a narrow focus on conventional (non-recycled) materials, absence of comparative hydraulic evaluation for recycled composites, and insufficient integration of hydraulic data with advanced predictive models for discharge optimization.

The study aims to experimentally assess and model the discharge behavior of recycled porous irrigation pipes: Type A (rubber-polyethylene blend) and Type B (recycled rubber). An Enhanced MLP deep learning model will be used for discharge prediction. Specifically, we will quantify differences in hydraulic performance, including discharge rate, coefficient of variation (CV), and emission uniformity (EU), between the pipe types under varying pressures (20–80 kPa) and lengths (3–9 m). Additionally, we will develop and benchmark four deep learning models- Enhanced MLP, LSTM, DNN, and ANN—employing synthetic data augmentation to improve model robustness. Finally, we propose a practical framework for integrating predictive analytics into irrigation scheduling in water-scarce regions. By bridging material science, hydraulics, and artificial intelligence, this work advances novel strategies for sustainable water management.

## Materials and methods

### Experimental location

The laboratory experiments were conducted at the Irrigation and Hydraulic Laboratory, Water and Irrigation Systems Engineering Department, Faculty of Agricultural Engineering, Al-Azhar University, Cairo, Egypt (30° 03′ 15.9″ N, 31° 19′ 14.9″ E).

### Porous pipes

Two types of porous pipes were evaluated: Type A, made from a blend of recycled rubber and polyethylene, manufactured by INNARI Innovation Nature Irrigation GmbH, 2019, and Type B, made from recycled rubber only, manufactured by Ecotube Germany GmbH, 2022. Both types had an outer diameter of 0.016 m with equal length and wall thickness of 9 m and 0.002 m, respectively. The pipes were produced in Germany and characterized by their coarse black surfaces with micro-pore structures^39,40^.

### Experimental design

**Fig. 1:**
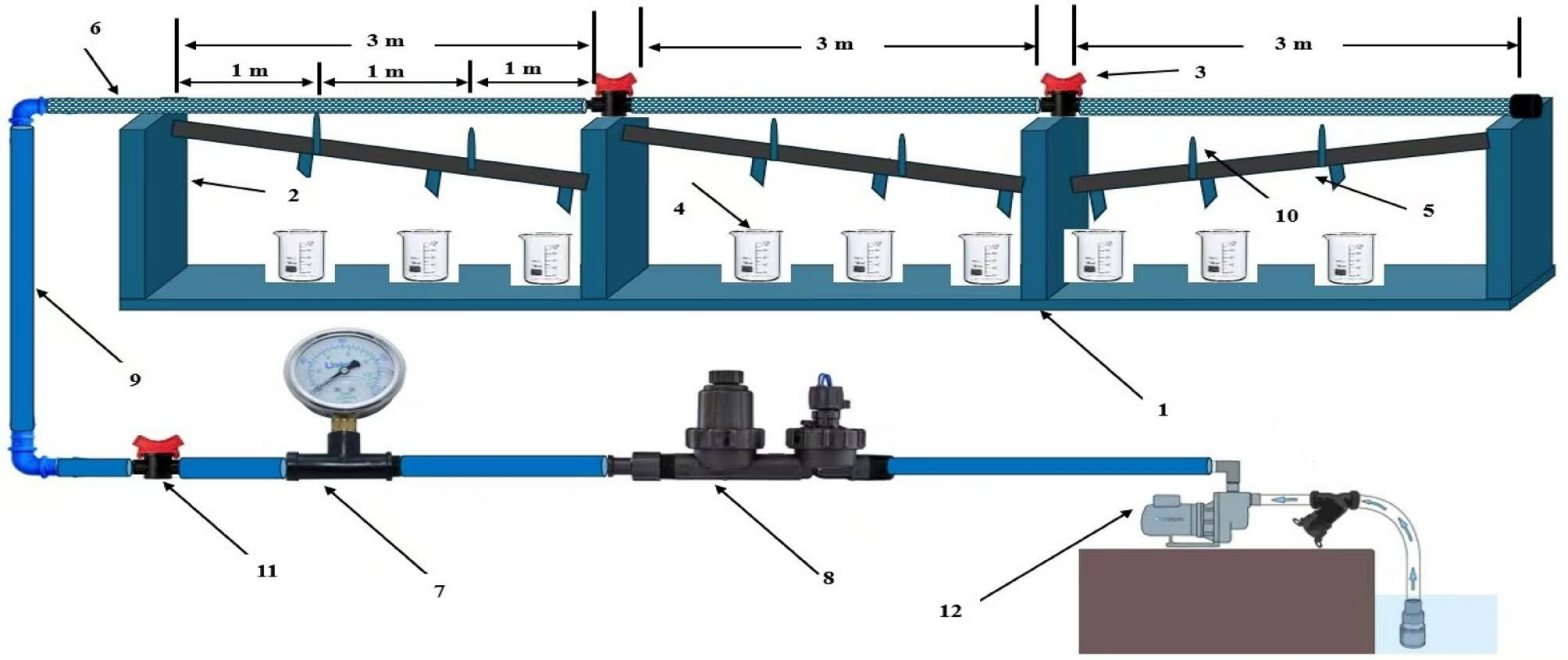
Experimental Design. 1—Wood frame. 2—Wooden stand. 3—Valve. 4—Container (Beaker). 5—PVC pipe ɸ 0.050 m. 6—Porous pipe ɸ 0.016 m. 7—Manometer. 8—Disk filter 120 mesh. 9—Flixable hose, 0.016 m. 10—Barrier. 11—Valve. 12—Pump.

The hydraulic performance of two porous pipe types (Type A and Type B) was assessed using a completely randomized design (CRD) with a split-plot layout. The experiments were conducted under consistent conditions, testing four operating pressures (20, 40, 60, and 80 kPa) and three pipe lengths (3, 6, and 9 m), as detailed in Fig. 1. Pressure was precisely regulated using a manual 16 mm control valve and monitored in real-time with a calibrated analog pressure gauge. Each combination of pressure and length was replicated three times, resulting in 72 experimental units. This replication strategy adheres to ASABE standards (EP458.1) for irrigation component testing, ensuring statistically robust estimation of variance and confidence intervals (95% CI) for hydraulic parameters. The porous pipes were positioned horizontally on a water collection system, designed with barriers at 1 m intervals to capture the flow from each section separately. The entire setup included a 9 m pipe length divided into three 3 m sections connected by diameter ($\varnothing$) 0.016 m valves, ensuring accurate flow measurement along each segment. A continuous water supply was provided using a pump, and flow rates were recorded using graduated cylinders (500 ml capacity), stopwatches, and nine collection containers placed at 1 m intervals. This method allowed precise measurement of discharge rates and facilitated the evaluation of emission uniformity across varying pressure levels and pipe lengths.

### Deep learning algorithm

Machine learning is a data analysis method that enables computers to identify patterns and make decisions with minimal human involvement by extracting information from data. As a subfield of artificial intelligence, it equips computers with the capacity to learn from examples rather than relying on explicit programming instructions^28^. Ahmed et al.^26^ provided a comprehensive review of machine learning applications across various agricultural activities. Deep learning represents a specialized branch of machine learning, distinguished by its use of artificial neural networks with multiple hidden layers the term “deep” specifically refers to the depth of these networks. Each successive layer in a deep learning architecture converts input data into increasingly abstract representations that the following layers can use for prediction tasks. These deep learning models have grown in popularity in recent years due to their faster and more efficient performance than classic machine learning models, as well as their ability to automatically extract significant features from raw data without requiring extensive preprocessing.

### Deep learning model development

Four deep learning models- Enhanced MLP, LSTM, DNN, and ANN were selected to predict porous pipe discharge. These models were chosen to facilitate a performance comparison based on their diverse architectures, proven accuracy, deep learning capabilities, implementation flexibility, and overall predictive power. These models offer different architectural approaches to exploring patterns in data, with Enhanced MLP demonstrating exceptional accuracy in capturing complex nonlinear relationships between pressure, pipe length, and material type, while LSTM provides time-series processing capabilities for future studies. Their deep neural network architectures enable the learning of complex data representations without extensive preprocessing, which is particularly beneficial for high-dimensional agricultural data. Implementation flexibility across software tools like TensorFlow and PyTorch facilitates their application in various environments, while the diverse set of models allows for fair performance comparison to identify the optimal model for practical irrigation management applications. Models were evaluated using R-squared, MAE, RMSE, and Test Loss metrics.

#### Enhanced MLP model architecture for predictive irrigation discharge modeling

**Fig. 2:**
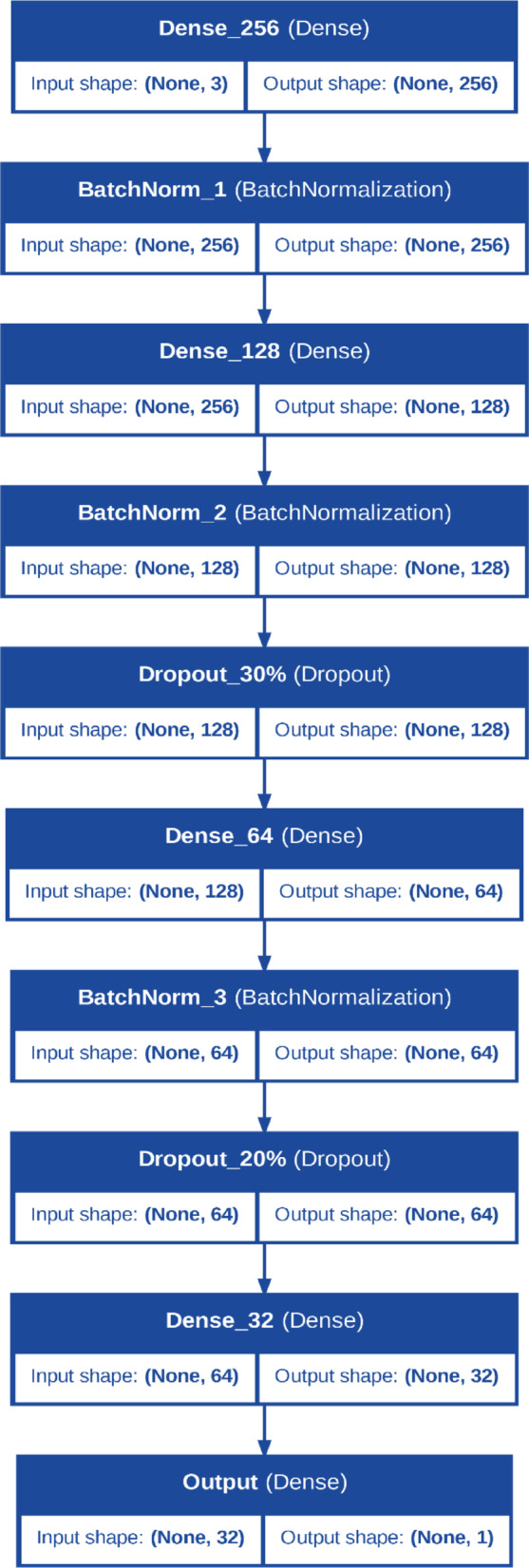
Enhanced MLP architecture model.

The Enhanced MLP model, shown in Fig. 2, is designed to predict discharge rates in porous pipe irrigation systems. Its architecture includes multiple dense layers with ReLU activation functions, batch normalization for stable training, and dropout layers (0.2–0.3) to prevent overfitting. The model accepts inputs such as operational pressure, pipe length, and material type. Implemented using TensorFlow and Keras, it benefits from optimized computational graphs. The training utilized the Adam optimizer and early stopping.

#### Architecture comparison of deep learning models for porous pipe discharge prediction

Table 1 shows the Architectural Features of Deep deep-learning models.

**Table 1:** Architectural features of deep learning models.

Model	Architecture type	Number of layers	Neurons per layer	Activation functions	Regularization techniques
Enhanced MLP	Feedforward NN	7	256, 128, 64, 32	ReLU	Batch normalization, Dropout
LSTM	Recurrent NN	3	50 (LSTM), 25, 1	ReLU, Linear	None
DNN	Feedforward NN	7	256, 128, 64, 32	ReLU	Dropout
ANN	Feedforward NN	5	128, 64, 32	ReLU, Linear	Dropout

#### Software and tools used

This research was predominantly executed utilizing Google Colab, a cloud-based development platform that offers complimentary access to high-performance computing resources, including GPUs and TPUs. Google Colab facilitated the implementation and training of the deep learning models by offering Python 3.11 as the primary programming language, a Jupyter Notebook interface for interactive code execution and results visualization, and accelerated training through GPU access. The seamless integration with Google Drive ensured efficient data storage and model saving throughout the development process.

### Data splitting and preprocessing

The experimental dataset was divided into training (70%) 2640, validation (15%) 566, and test (15%) 566 subsets using stratified random sampling to maintain a proportional representation of pipe types and pressures. Preprocessing steps, including normalization, were applied independently to each subset using training-set-derived parameters to prevent data leakage. Input variables (pressure, pipe length, pipe type) and discharge outputs were normalized to a [0, 1] range using MinMax scaling. This preprocessing step was applied independently to training, validation, and test sets using parameters derived solely from the training data to prevent leakage.The validation set monitored overfitting during hyperparameter tuning, while the test set evaluated the final model performance.

#### Key libraries and frameworks

**Fig. 3:**
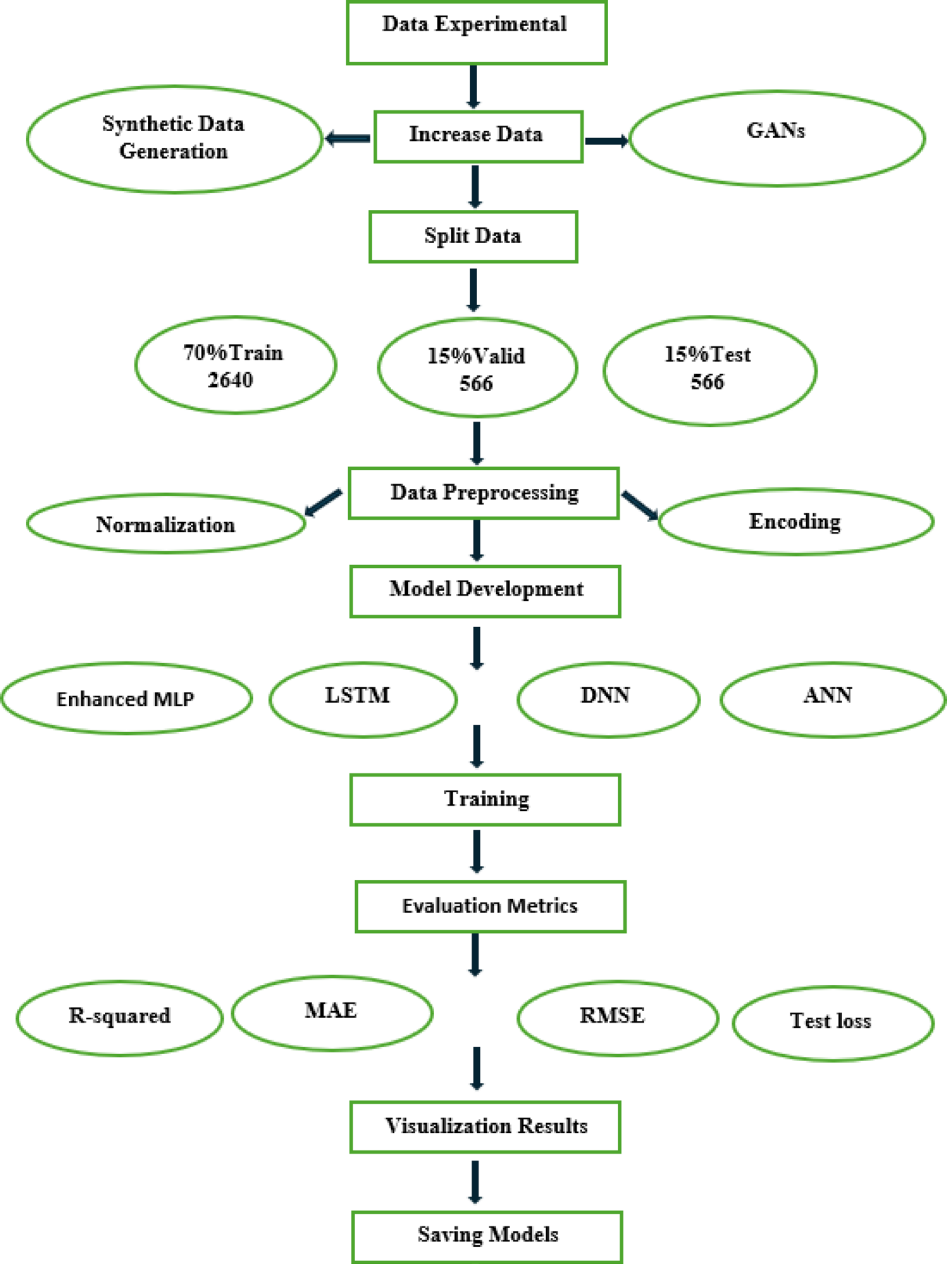
Workflow of deep learning model training and validation for porous pipe discharge prediction.

The workflow presented in Fig. 3 for training the porous pipe discharge prediction and validating deep learning models includes several important libraries and frameworks. The process begins with experimental data collection, followed by a crucial data augmentation step where GANs are employed to increase the dataset size from 72 to 3372 samples, significantly enhancing both data quality and quantity for model training^47^. Libraries such as Pandas and Scikit-learn, which are frequently used for data preprocessing, include normalization and encoding. The model development phase includes the activation of various types of neural network architectures, such as MLP, LSTM, DNN, and ANN, that can be realized by using TensorFlow. These frameworks provide the necessary tools to build, train, and evaluate complex neural networks. After training, evaluation metrics such as R-squared, Mean Absolute Error (MAE), Root Mean Square Error (RMSE), and test loss are calculated, which can be done through Scikit-learn or directly within TensorFlow or PyTorch. Eventually, the Matplotlib and Seaborn libraries can be used for data visualization, followed by the saved trained models, which are often saved with the respective functions provided by TensorFlow.

## Training parameters

Table 2 summarizes the training configurations for the four deep learning models evaluated in this study.

**Table 2:** Training parameters of deep learning models.

Model	Regularization techniques	optimizer	Training parameters
Enhanced MLP	L2 regularization (0.001), dropout (0.3, 0.3, 0.2)	Adam (lr = 0.0005)	500 epochs, batch size 32, validation on validation set
LSTM	L2 regularization (0.001), dropout (0.3, 0.3, 0.2)	Adam (lr = 0.0005)	500 epochs, batch size 32, validation on validation set
DNN	Dropout (0.3, 0.3, 0.2)	Adam (lr = 0.0005)	500 epochs, batch size 32, validation on validation set
ANN	Dropout (0.2, 0.2)	Adam (lr = 0.0005)	500 epochs, batch size 32, validation on validation set

### Measurements

#### Pressure–discharge relationship

The relationship between discharge and pressure was modeled using a power function, as expressed in Eq. (1) ^35,41^:

1 $$q = kp^{x}$$

where q is the flow rate (L/h/m), k is the emitter constant, the operating pressure (kPa), and x is the emitter exponent. This power relationship characterizes the flow regime of irrigation emitters, where an exponent value of 1 indicates laminar flow, 0.5 denotes fully turbulent flow, and intermediate values signify partially turbulent flow^42,43^. Values below 0.5 suggest pressure-compensating properties.

#### Coefficient of variation (CV)

The coefficient of variation is the ratio of the standard deviation of the mean flow to the mean flow across a sample of emitters. It is calculated according to Eq. (2) ^33,44^.

2 $$C_{V} = \frac{s}{q}$$

where $C_{V}$ is the manufacturing coefficient of variation, q is the average emission rate of the sample, and S is the sample standard deviation.

3 $$S = \sqrt {\frac{1}{n - 1}} \mathop \sum \limits_{i = 1}^{n} \left( {q_{i} - q_{a} } \right)^{2}$$

where n is the number of linear meters of porous pipes, $q_{i}$ is the porous pipe flow rate (L/h/m) and $q_{a}$ is the average of porous pipe flow rates (L/h/m).

According to ASAE standard 2019^45^, a variation greater than 20% is considered unacceptable for line source emitters, while a variation less than 10% is deemed good, and variations between 10 and 20% are considered acceptable.

#### Emission Uniformity (EU)

Emission uniformity for point and line source emitters was defined using Eq. 4 proposed by^46^:

4 $${\text{EU}} = {1}00\left( {{1} - \frac{1.27}{{\sqrt {N_{e} } }} C_{V} } \right)\frac{{q_{min} }}{{q_{ave} }}$$

where EU is the design emission uniformity in percent, N_e_ is the Number of line source porous pipe per emission point, $C_{V}$ *Is the* manufacturer’s coefficient of variation for line source porous pipe, $q_{min}$ is the minimum porous pipe discharge rate in the system (L/h/m), $q_{ave}$ is the average or porous pipe discharge rate (L/h/m).

#### Statistical analysis

The statistical analysis was conducted using Statistix model 8.1, applying a factorial analysis of variance (ANOVA) at a 5% significance level to evaluate the effects of pipe type, pressure, and length on discharge rates. A factorial layout was applied, especially a 2 × 4 × 3 factorial design. In this layout, the primary issue (pipe kind) has 2 ranges (Type A and Type B), the second issue (running pressure) has 4 ranges (20, 40, 60, and 80 kPa), and the third factor (pipe length) has 3 tiers (3, 6, and 9 m). Each aggregate of factors was replicated in 3 instances, resulting in a total of 72 experimental devices (2 × 4 × 3 × 3 = 72). The experimental units were arranged in a Completely Randomized Design with a Split-Plot Design.

#### Deep learning models evaluation

Models were evaluated using R-squared, MAE, RMSE, and Test Loss metrics. RMSE and R^2^-score are calculated by Eqs. (5) and (6), respectively:

5 $${\text{RMSE}} = \sqrt {\frac{{\mathop \sum \nolimits_{i = 1}^{n} \left( {y_{i} - {\acute{y}}_{i} } \right)^{2} }}{n}}$$

6 $${\text{R}}^{{2}} = {1} - \frac{{\mathop \sum \nolimits_{i = 1}^{n} \left( {y_{i} -{\acute{y}}_{i} } \right)^{2} }}{{\mathop \sum \nolimits_{i = 1}^{n} \left( {y_{i} - y^{\prime}} \right)^{2} }}$$

where y, and $y^{\prime}$ are the true value, the predicted value, and the mean of the true values, respectively.

#### Using synthetic data generation technology (GANs) to increase data volume

The use of synthetic data generation technology, particularly through Generative Adversarial Networks (GANs), represents a valuable approach for enhancing both the quality and quantity of data used in deep learning models. GANs consist of two neural networks: a generator that creates synthetic data from random noise, and a discriminator that evaluates whether the data is real or fake. These networks engage in a competitive process where the generator aims to produce increasingly realistic data while the discriminator strives to accurately distinguish between real and synthetic samples.

In this study, GAN technology was employed to increase the dataset from 72 to 3772 samples, providing several important benefits. First, improved model accuracy was achieved as the larger dataset enabled models to be trained on a more diverse set of scenarios, enhancing their ability to generalize and improving prediction accuracy^47^. With 3772 samples, models could identify more varied patterns in porous pipe discharge under different conditions. Second, synthetic data generation helped reduce bias by ensuring a comprehensive representation of all possible cases through controlled methods, mitigating potential limitations in the original dataset or underrepresentation of certain scenarios. Third, enhanced training efficiency was observed as models were exposed to more examples during the same number of training epochs, accelerating learning and performance improvement^47^. Furthermore, the generated synthetic data mimicked real-world operating conditions that might be encountered in the field, making models more applicable to diverse agricultural environments and varying. Finally, this approach provided economic and sustainable benefits by avoiding the need for additional costly laboratory experiments, thus providing an economical way to expand the dataset while maintaining data quality^48^. Although input variables (pressure, length, pipe type) are low-dimensional, GAN augmentation was scientifically justified due to the complex nonlinear sensitivity of discharge to interacting parameters and the prohibitive costs of exhaustive physical experimentation across real-world operational scenarios. Synthetic data preserved empirically derived physical relationships (section “Effect of pressure on the discharge of porous pipes”, Table 3) while expanding coverage of parameter interactions beyond laboratory constraints.

**Table 3:** Developed models for the pressure discharge relationship for two types of porous pipes.

Porous type		k	x	Developed model	Goodness of fit (R^2^)	Classification
Type A	ql3	0.982	0.5459	ql3 = 0.982p^0.5459^	0.9612	Fully turbulent flow
	ql6	0.5163	0.6623	ql6 = 0.5163p^0.6623^	0.9772	Mostly turbulent flow
	ql9	0.4855	0.6596	ql9 = 0.4855p^0.6596^	0.9645	Mostly turbulent flow
Type B	ql3	0.4608	0.8424	ql3 = 0.4608p^0.8424^	0.9873	Mostly laminar flow
	ql6	0.3899	0.8519	ql6 = 0.3899p^0.8519^	0.9846	Mostly laminar flow
	ql9	0.2868	0.9081	ql9 = 0.2868p^0.9081^	0.9899	Mostly laminar flow

## Results and discussion

### Effect of pressure on the discharge of porous pipes

**Fig. 4:**
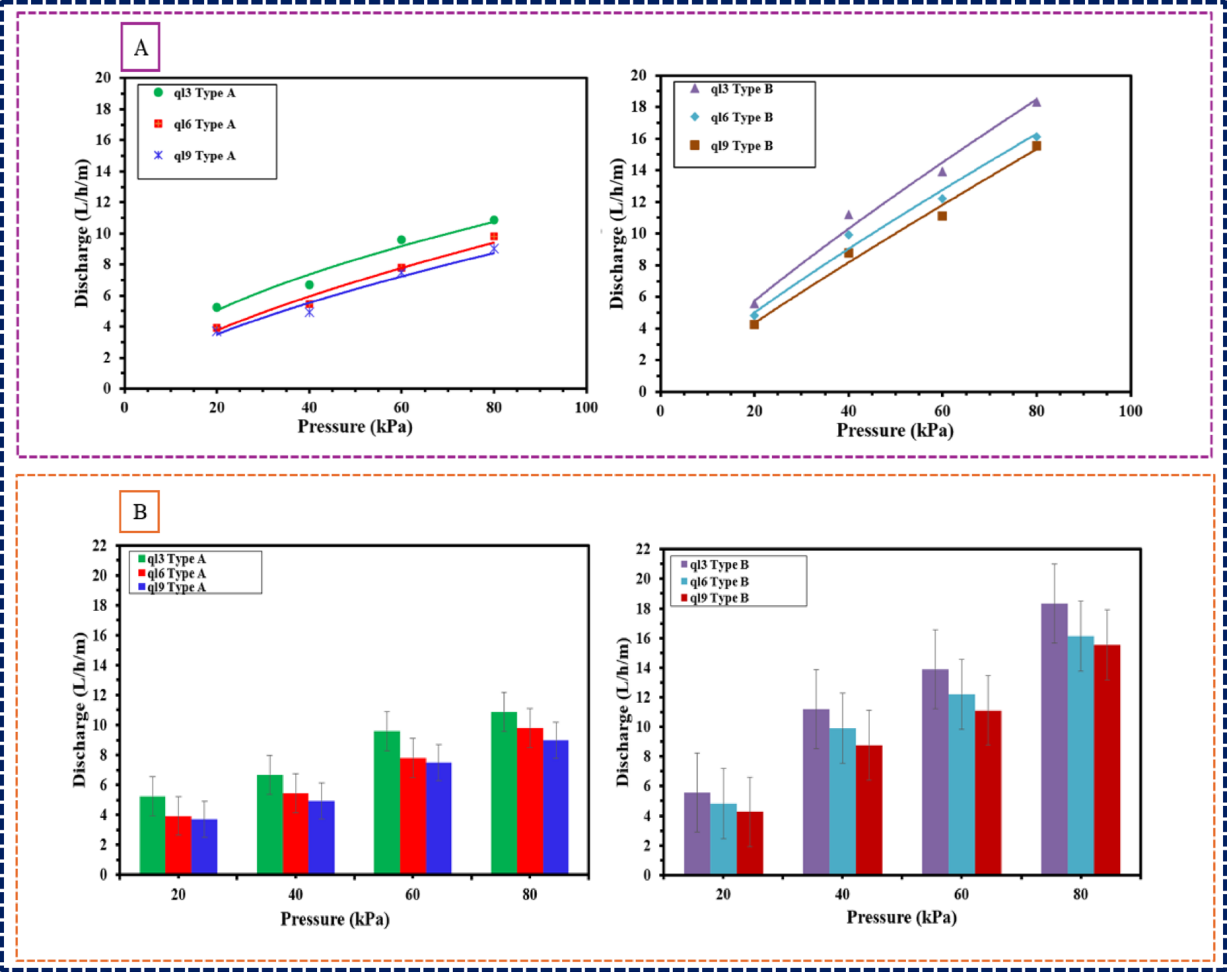
Relationships between the discharge of two types (A, B) for porous pipe and operating pressure.

The relationship between pressure and discharge in porous pipes is illustrated in Table 3 and Fig. 4A and B The discharge values represent average measurements obtained from each pipe during the initial 20 min of testing under varying pressure conditions. Regression analysis indicated a strong correlation between discharge and pressure, with coefficients of determination R^2^ of 0.95 and 0.97 for Type A and Type B pipes, respectively.

The constants K and x vary by pipe type and measurement units. Discharge significantly changes with pressure, highlighting its influence. The different discharge-pressure exponents (0.54–0.66 for Type A, 0.8–0.9 for Type B) reflect material differences, with Type B showing higher sensitivity due to its rubber composition. These findings indicate that increased operating pressure substantially increases emission rates, Type B shows higher discharge compared to Type A under the same conditions, indicating greater sensitivity to pressure changes. Additionally, the error bars illustrate the confidence level in the data, with results appearing consistent and low measurement variance. The higher exponents for Type B suggest a more pronounced laminar flow regime. Type B’s superior sensitivity (exponent 0.8–0.9) stems from recycled rubber’s homogeneous microstructure, which maintains stable pore geometry under pressure, promoting laminar flow. Conversely, Type A’s rubber-polyethylene blend exhibits localized stress concentrations under load, causing non-uniform pore deformation and turbulent flow (exponent 0.54–0.66). The rubber tire material likely facilitates smoother flow characteristics, reducing turbulence compared to the composite material of Type A’s turbulent flow regime.

This difference in behavior aligns with previous studies, where emitters made from non-elastic materials like polyethylene (PE) and polyvinyl chloride (PVC) typically exhibit discharge-pressure exponents ranging from 0.5 to 0.8. The observed flexibility of Type A pipes, attributed to the polyethylene component, contributed to enhanced hydraulic performance by increasing flow velocity through each emission pore, enlarging pore dimensions, and creating additional effective emission pores as pressure increases This adaptability may account for the predominantly turbulent flow regime observed in Type A, suggesting a more complex interaction between pressure and discharge in these pipes. These results are consistent with previous studies by^25,33,34,36,49,50^ Those who reported that recycled rubber pipes typically exhibit smoother flow characteristics due to their homogenous structure. and uniform materials reduce internal turbulence, leading to more efficient water transport.

Furthermore, the classification of flow regimes for both types of porous pipes aligns with established guidelines that correlate the emitter discharge exponent with the nature of the flow regime^42,43^. Understanding these flow dynamics is critical for optimizing the design and application of porous irrigation systems in agricultural practices.

ANOVA confirmed that pressure, length, and material significantly affect porous pipe discharge (*p* < 0.05). Strong pressure-discharge correlation (R^2^ = 0.95–0.97) was found, with Type B pipes showing higher sensitivity due to laminar flow characteristics. The statistical analysis supports these observations, as pressure interaction terms significantly affect discharge rates.

### Effect of length on the discharge of porous pipes

The relationship between porous pipes’ length and discharge rates is a critical determinant of irrigation efficiency and system design. This study investigated two porous pipe types (Type A and Type B) across various lengths designated as ql3, ql6, and ql9. The discharge rates for each type were meticulously measured and analyzed to elucidate how pipe length influences overall hydraulic performance, as illustrated in Table 3 and Fig. 4A and B.

For Type A pipes, the discharge rates in L/.h/ m exhibited a clear decreasing trend with increasing length: ql3 (5.24), ql6 (3.92), and ql9 (3.68) L/.h/ m. This trend indicates that as the pipe length increases, the resistance to flow also rises, resulting in diminished discharge rates.

The discharge rates for Type B pipes for ql3, ql6, and ql9 at 20 kPa were 5.56, 4.82, and 4.26 (L/h/m), respectively. Like Type A, Type B decreases in discharge rates with increased pipe length. However, as the operating pressure rises from 20 to 80 kPa, the discharge rates for all lengths significantly improve, reaching 18.32, 16.12, and 15.54 (L/h/m) for ql3, ql6, and ql9, respectively, at 80 kPa. This suggests that higher operating pressures can effectively mitigate the adverse effects of increased pipe length on discharge rates.

The difference in performance may be attributed to the material composition of Type A, which may offer enhanced flexibility and lower frictional resistance due to the polyethylene content. The comparative analysis of discharge rates between the two types indicates that Type B pipes demonstrate greater resilience to changes in length, particularly under elevated pressures. The flow dynamics observed in Type B pipes can be attributed to their specific design and material properties, which likely facilitate a more efficient water transport mechanism despite the challenges posed by increased length. These results are consistent with the findings of^36^, who reported that porous pipes made from recycled rubber demonstrated better flow characteristics under increasing length and pressure.

ANOVA confirms pipe length significantly affects discharge, with both types showing decreased rates as length increases. The interaction between pressure, length, and material is statistically significant, highlighting the importance of material properties. Type A’s turbulent flow offers more adaptability. Designing irrigation systems requires careful consideration of both pipe length and material to optimize performance and efficiency, as longer pipes may need higher pressure, increasing energy costs.

### The effect of pressure on the coefficient of variation (CV)

**Fig. 5:**
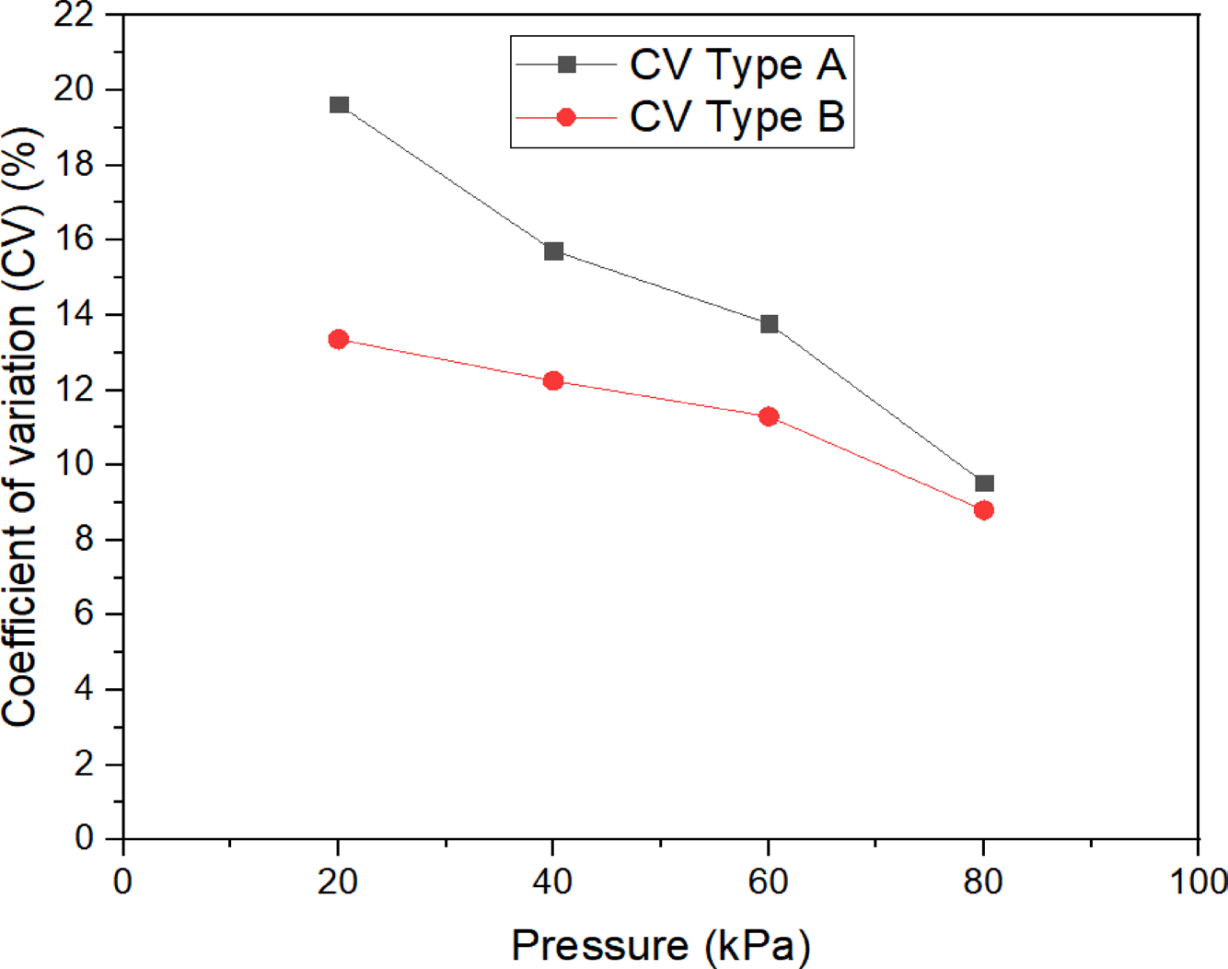
Relationships between the coefficient of variation (CV) of two types (A, B) for porous pipe and operating pressure.

The influence of operating pressure on the coefficient of variation (CV) for both types of porous pipes is illustrated in Fig. 5. As the operating pressure increased, the CV decreased an initial decline for both types of pipes. In the 20–80 kPa pressure range, the CV values reached their lowest values of 9.54% for Type A and 8.80% for Type B, both at the 80 kPa value. These results suggest that the homogeneity of discharge and emission rates for the porous pipes are highly sensitive to pressure variations, which means that pressure must be a crucial consideration in evaluating discharge effectiveness.

According to the ASAE Standard 2019^45^, a CV of less than 10% is classified as excellent, a CV ranging from 10 to 20% is deemed acceptable, while a CV exceeding 20% is considered unacceptable for line-source emitters. In the present investigation, the CV values for both Type A and Type B pipes throughout the 20–60 kPa pressure range remained within acceptable parameters, demonstrating commendable uniformity at 80 kPa for both categories.

The variations in material composition among the pipes likely impacted the observed results. Type A pipes, constructed from a composite of recycled rubber and polyethylene, may leverage the flexibility of polyethylene, which enhances flow dynamics and facilitates a more uniform discharge across varying pressures. This flexibility could elucidate the lower CV recorded for Type A pipes, particularly under higher pressure scenarios, as it enables the pipe to more effectively accommodate variations in hydraulic conditions. Conversely, Type B pipes, fabricated entirely from recycled rubber, exhibited marginally lower CV values overall. This improved uniformity arises from rubber’s viscoelastic properties, which dampen pressure-induced vibrations and ensure consistent flow paths. Type A’s composite interface creates micro-turbulence, increasing flow variability. This observation suggests that the homogeneous material structure of Type B pipes provides more stable flow characteristics in response to pressure variations compared to Type A’s composite nature. These results are consistent with previous studies by^25,33,36,49,50^.

### Emission uniformity of porous pipes

**Fig. 6:**
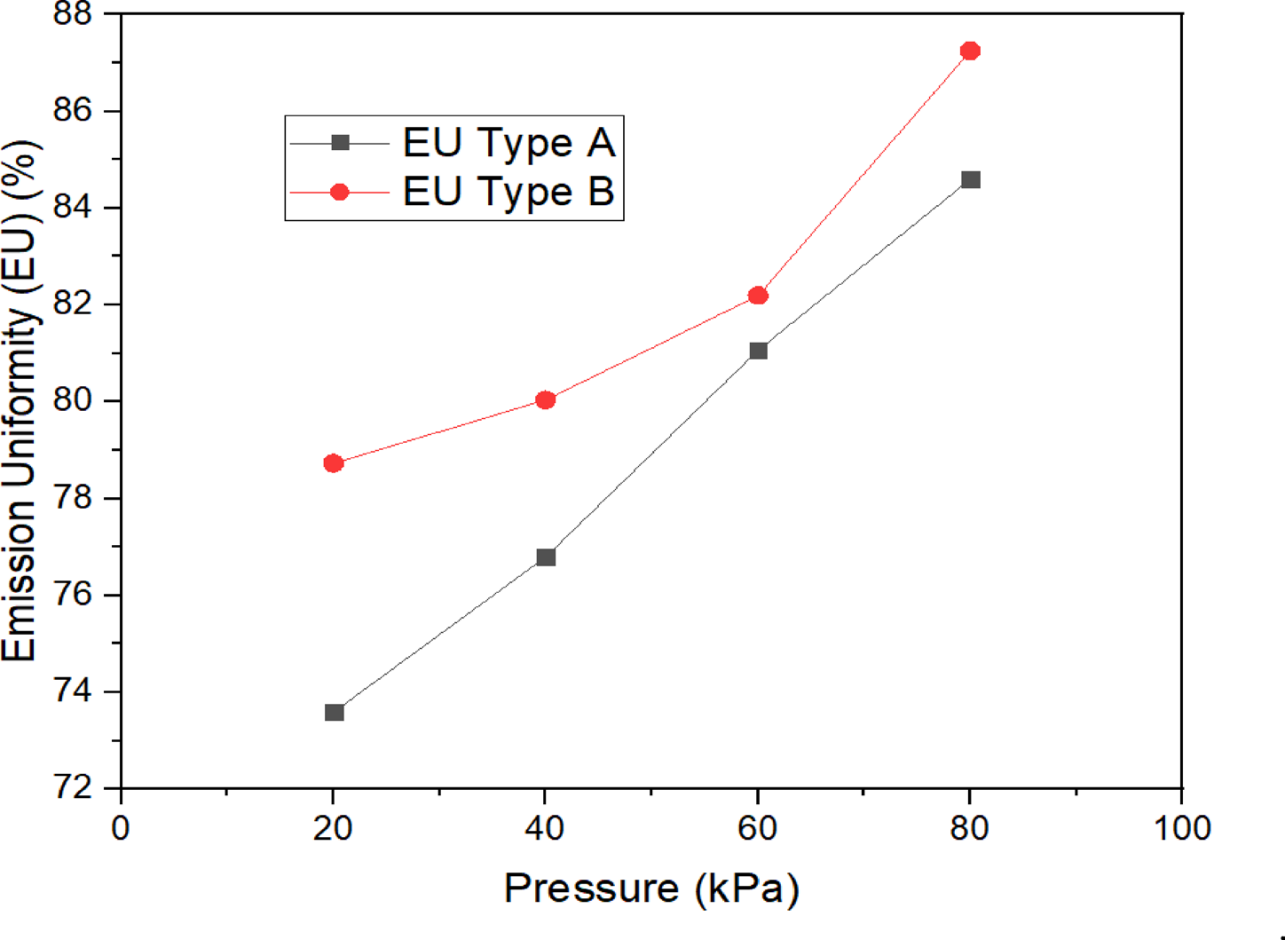
Relationships between the emission uniformity (EU) of two types (A, B) for porous pipe and operating pressure.

For Type A porous pipes, the data illustrated in Fig. 6 elucidate that the emission uniformity values fluctuated between 73.59 and 84.60% across operational pressures ranging from 20 to 80 kPa. The ASAE classification standards were established in 2019^45^. The functionality of Type A pipes was classified as fair at both 20 and 40 kPa and as good at pressures spanning from 60 to 80 kPa. This observation implies that while Type A pipes demonstrate adequate emission uniformity at elevated pressures, Type B porous pipes exhibited emission uniformity values ranging from 78.83 to 87.25% within the identical pressure interval. According to the ASAE classification, the performance of Type B pipes was assessed as fair at 20 kPa and classified as good at 40, 60, and 80 kPa.

The better uniformity of Type B pipes can be attributed to their homogeneous rubber material, which provides stable flow dynamics. The variability in Type A pipes may be due to the polyethylene blend, which introduces additional flow resistance and increases variability under lower pressure conditions. These observations align with previous studies by^50^, who reported that homogeneous materials tend to exhibit more consistent emission rates. Material differences explain performance gaps: Type A’s flexibility may cause emission variations, while Type B’s rigidity ensures more stable flow and uniformity.

### Deep learning model evaluation

**Fig. 7:**
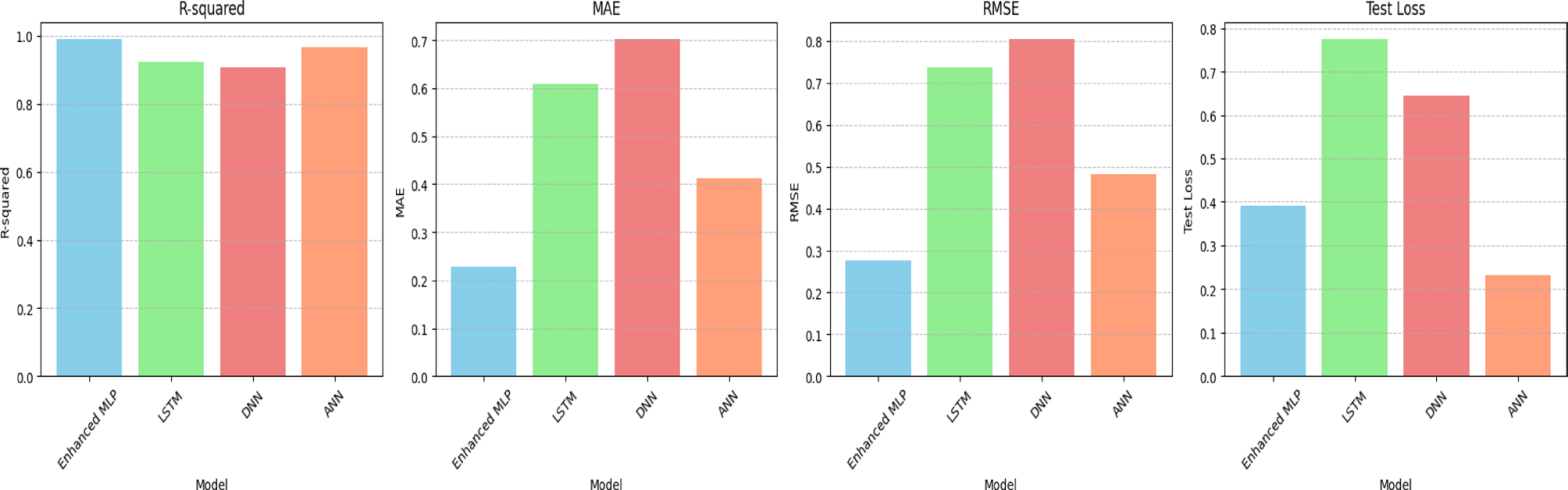
Evaluation of performance metrics of Deep Learning Models to Predict discharge of porous pipes.

**Fig. 8:**
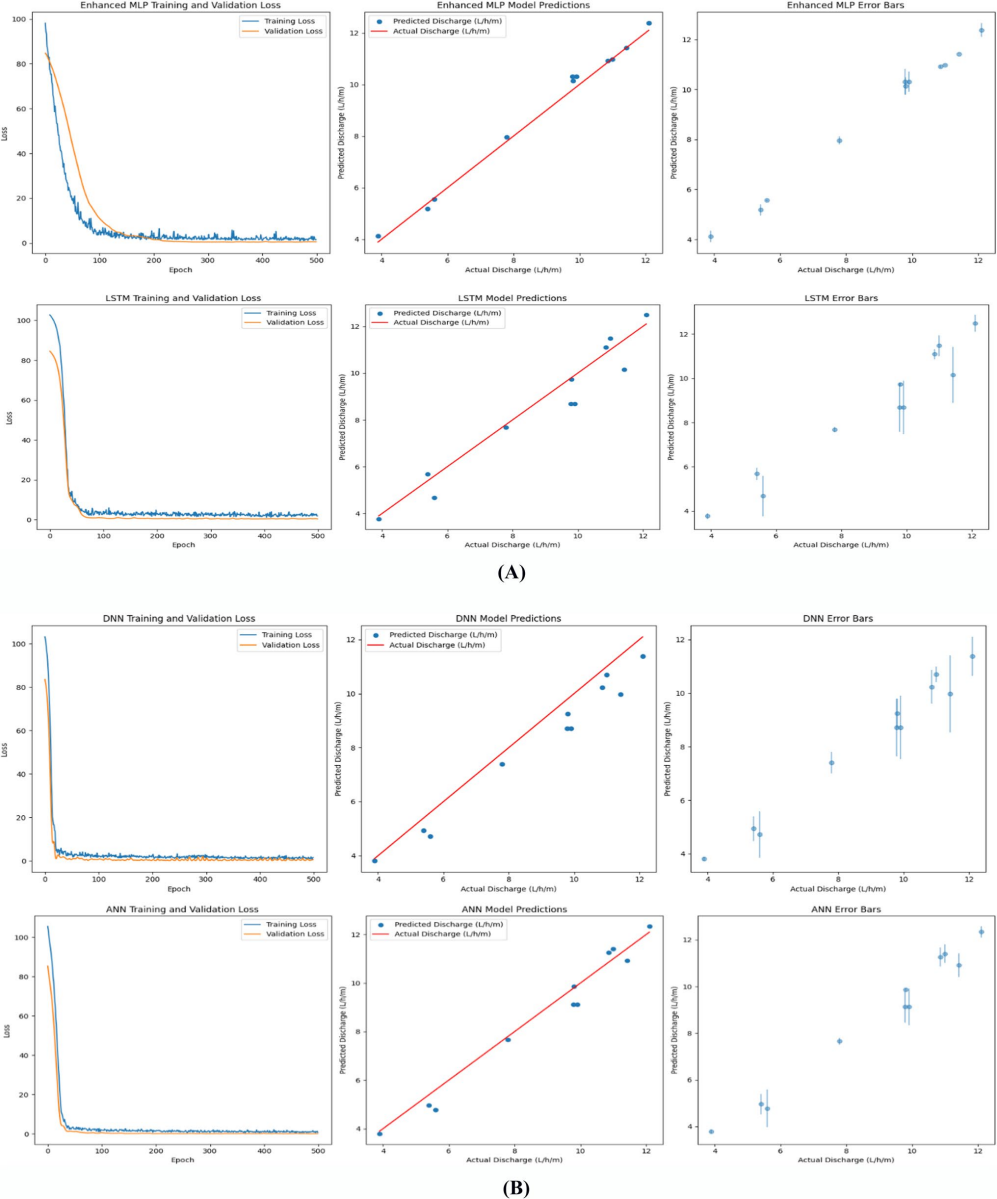
(**A**) Performance comparison of deep learning models for porous pipe discharge prediction. (**B**) Performance comparison of deep learning models for porous pipe discharge prediction.

The results presented in Table 4 and visualized in Figs. 7 and 8A and B provide a comprehensive comparison of the evaluation of four deep-learning models for predicting porous pipe discharge rates, revealing significant performance differences. The Enhanced MLP model demonstrated superior accuracy with an R-squared value of 0.9891, MAE of 0.2286, RMSE of 0.2762, and test loss of 0.3923. The term ‘Enhanced’ denotes three critical modifications over standard MLPs: (1) batch normalization between layers to accelerate convergence and reduce sensitivity to initialization; (2) targeted dropout (0.2–0.3) on high-dimensional layers (256–128 neurons) to prevent co-adaptation; and (3) L2 regularization (λ = 0.001) to constrain weight magnitudes. This architecture specifically counters overfitting in small agricultural datasets while capturing nonlinear interactions—advantages absent in LSTM (overfits non-temporal data), DNN (lacks normalization), and ANN (shallow representation). In comparison, the LSTM, DNN, and ANN models showed progressively lower performance across these metrics, with the DNN model performing least effectively (R-squared = 0.9077, MAE = 0.7016, RMSE = 0.8036, test loss = 0.6458).

**Table 4:** Evaluation performance metrics of deep learning models.

Model	R-squared	MAE	RMSE	Test loss
Enhanced MLP	0.9891	0.2286	0.2762	0.3923
LSTM	0.9225	0.6084	0.7364	0.7740
DNN	0.9077	0.7016	0.8036	0.6458
ANN	0.9667	0.4124	0.4823	0.2326

The Enhanced MLP’s architecture, featuring multiple dense layers with ReLU activation, batch normalization, and strategic dropout layers, proved particularly effective at capturing the complex relationships in the irrigation data. This model’s exceptional accuracy makes it highly suitable for real-world irrigation management applications where precise water scheduling and system optimization can lead to significant water savings and improved crop yields, These results align with previous research highlighting MLP’s effectiveness in complex agricultural data analysis^28^. While our findings confirm that Enhanced MLP is the optimal model for predicting porous pipe discharge under varying operating conditions, they also show that model selection should be based on specific data characteristics and application requirements. The lower performance of LSTM in this study (R^2^ = 0.9225) compared to Enhanced MLP likely stems from the non-temporal nature of our dataset, which contrasts with previous studies demonstrating LSTM’s effectiveness with time-series data^31^. Similarly, the moderate performance of DNN (R^2^ = 0.9077) suggests that increased network depth does not necessarily lead to performance improvements without corresponding data complexity, consistent with^26^ observation that network depth should be carefully considered relative to data characteristics. The ANN model showed interesting trade-offs with lower test loss despite higher MAE and RMSE, suggesting good generalization capabilities that might be valuable in specific contexts with which these results agree^27^.

These findings confirm Enhanced MLP as the optimal model for predicting porous pipe discharge under varying conditions.

### Performance comparison of deep learning models for porous pipe discharge prediction

Figure 8A and B presents a comprehensive performance comparison of four deep learning models (Enhanced MLP, LSTM, DNN, and ANN) in predicting porous pipe discharge. The Enhanced MLP model consistently outperforms the other models across all evaluation metrics. Its training and validation loss curves demonstrate efficient learning with minimal overfitting, its predictions exhibit the highest accuracy with tight clustering around the actual values, and the error bars indicate the lowest prediction uncertainty. In contrast, the LSTM model shows the poorest performance, with the training and validation loss curves indicating significant overfitting, the predictions displaying the most scatter, and the error bars revealing the highest uncertainty. The DNN and ANN models exhibit intermediate performance levels. Both show signs of overfitting in their loss curves, with the ANN model showing a higher degree of overfitting than the DNN. Their predictive accuracy is inferior to that of the Enhanced MLP model, with error bars indicating elevated levels of uncertainty when compared to the Enhanced MLP. In summary, the Enhanced MLP architecture demonstrates exceptional proficiency in modeling the complex relationships within porous pipe discharge data. This highlights the critical importance of implementing customized architectural elements such as batch normalization and dropout techniques when developing models for this specific application, as these design choices significantly enhance predictive performance.

### Practical applications

The outcomes of this study significantly advance the improvement of irrigation systems, particularly in water-scarce regions and environmentally conscious agricultural practices. Type A pipes (recycled rubber and polyethylene) demonstrate high durability and suitability for permeable soils, providing a consistent water supply for sensitive crops such as vegetables and fruits. Type B pipes (recycled rubber only) exhibit superior water discharge uniformity, making them ideal for dryland agriculture. Both pipe types effectively reduce water waste and promote sustainable farming practices. Optimal performance across both pipe types is achieved at 80 kPa pressure, balancing water application efficiency with conservation goals.

The deep learning models developed in this study add substantial value to these practical applications. The Enhanced MLP model’s exceptional prediction accuracy (R^2^ = 0.9891) enables precise forecasting of discharge rates under varying operational conditions. This capability allows farmers and irrigation managers to:

- Optimize irrigation scheduling based on predicted water needs.
- Detect potential system issues before they cause water waste or crop stress.
- Implement precision agriculture techniques that maximize water use efficiency.
- Adapt quickly to changing environmental conditions or water availability.

## Conclusion

This study provides detailed insights into the hydraulic performance of recycled porous pipes and indisputably illustrates the efficacy of deep learning algorithms in precisely predicting discharge rates. The results showed that Type B pipes had better water distribution uniformity and pressure sensitivity than Type A pipes containing a polyethylene blend while Type B pipes had a coefficient of variation as low as 8.80% at 80 kPa, demonstrating outstanding homogeneity. The Enhanced MLP model outperformed the other deep learning architectures in terms of prediction accuracy, with an R-squared value of 0.9891 and an RMSE of 0.2762. The model’s great ability to anticipate discharge rates under a variety of situations.

These discoveries inform agricultural engineers and water resource managers about very important issues, particularly in arid regions, where quality irrigation is essential. The deep learning approach’s enhanced predictive capabilities would enable the design and operation of a more efficient irrigation system with the potential to reduce water waste significantly, while the crop yield would be preserved or even improved. This precision-driven approach supports the transition towards precision agriculture, optimizing resource use and minimizing environmental impact. The results also underscore the importance of informed material selection in porous pipe manufacturing for achieving optimal hydraulic performance and system longevity. Practically, the Enhanced MLP model can be deployed through cloud-based decision support systems that integrate IoT sensors (pressure, soil moisture) with weather forecasts. Farmers may access real-time discharge predictions via mobile applications, enabling dynamic irrigation scheduling. For automated systems, model outputs can directly control valve operations based on predicted water needs.

However, this study has limitations that warrant consideration. The experiments were conducted under controlled laboratory conditions, which may not fully encapsulate the complexities of diverse field environments, including variations in soil composition, temperature fluctuations, and wind effects. Future research should prioritize field validation of these models across a spectrum of agricultural conditions to rigorously assess their performance and robustness in real-world applications. Furthermore, exploring and developing hybrid deep learning architectures that synergize the strengths of different neural network types could potentially unlock even greater gains in prediction accuracy and model versatility. The development of user-friendly mobile applications and robust decision-support systems based on these advanced models would facilitate their widespread adoption by farmers and irrigation managers, thereby accelerating the implementation of sustainable water use practices in agriculture and contributing to global water security. We believe that the current study provides insights into the importance of considering the selection of porous pipes based on the materials used in their manufacturing due to their impact on the hydraulic performance in irrigation systems, especially in arid areas, and opens the way for further research to optimize the manufacturing of material components for these pipes and operating conditions to improve irrigation efficiency and sustainability. This work provides a template for merging material innovation with AI-driven optimization to advance sustainable water management in agriculture.

## Data Availability

Data available on request from corresponding author [mohamedahmed.17@azhar.edu.eg](mailto:mohamedahmed.17@azhar.edu.eg).

## Abbreviations

Type A: Porous pipes made of recycled rubber and polyethylene
Type B: Porous pipes made of Recycled Rubber
P: Operating pressure (kPa)
ql3: Average of porous pipes discharge over 1 to 3 m of the pipe length (L/h/m)
ql6: Average of porous pipes discharge over 4 to 6 m of the pipe length (L/h/m)
ql9: Average of porous pipes discharge over 7 to 9 m of the pipe length (L/h/m)
CV: The manufacturer’s coefficient of variation for line source porous pipes (%)
EU: The design emission uniformity (%)
